# Genetic evidence of female kin clusters in a continuous population of a solitary carnivore, the Eurasian lynx

**DOI:** 10.1002/ece3.4562

**Published:** 2018-10-17

**Authors:** Katja Holmala, Annika Herrero, Alexander Kopatz, Julia Schregel, Hans G. Eiken, Snorre B. Hagen

**Affiliations:** ^1^ Natural Resources Institute Finland (Luke) Helsinki Finland; ^2^ NIBIO – Svanhovd Norwegian Institute of Bioeconomy Research Svanvik Norway

**Keywords:** genetic relatedness, kinship, *Lynx lynx*, matrilineal assemblage

## Abstract

Large terrestrial carnivores can sometimes display strong family bonds affecting the spatial distribution of related individuals. We studied the spatial genetic relatedness and family structure of female Eurasian lynx, continuously distributed in southern Finland. We hypothesized that closely related females form matrilineal assemblages, clustering together with relatives living in the neighboring areas. We evaluated this hypothesis using tissue samples of 133 legally harvested female lynx (from year 2007 to 2015), genotyped with 23 microsatellite markers, and tested for possible spatial genetic family structure using a combination of Bayesian clustering, spatial autocorrelation, and forensic genetic parentage analysis. The study population had three potential family genetic clusters, with a high degree of admixture and geographic overlap, and showed a weak but significant negative relationship between pairwise genetic and geographic distance. Moreover, parentage analysis indicated that 64% of the females had one or more close relatives (sister, mother, or daughter) within the study population. Individuals identified as close kin consistently assigned to the same putative family genetic cluster. They also were sampled closer geographically than females on average, although variation was large. Our results support the possibility that Eurasian lynx forms matrilineal assemblages, and comparisons with males are now required to further assess this hypothesis.

## 1 INTRODUCTION

Large terrestrial carnivores with high rates of dispersal and long‐distance movement usually also have high rates of gene flow among populations if dispersing individuals succeed to reproduce (Wayne & Koepfli, 1996). Even with highly mobile species, discontinuous habitat and anthropogenic‐associated barriers, such as major roads, monoculture, and human‐caused mortality, may constrain dispersal and reduce population densities (Loxterman, 2011; Sinclair et al., 2001; Walker, Harveson, Pittman, Tewes, & Honeycutt, 2000; Woodroffe, 2000). Accordingly, fragmentation and genetic drift may cause genetic substructuring in the population, for example gray wolf (*Canis lupus*) and Florida black bear (*Ursus americanus floridanus*) (e.g., Dixon et al., 2007; Vilà et al., 1999; Wayne, Lehman, Allard, & Honeycutt, 1992). In continuous populations, unhampered by fragmentation or isolation, dispersal and gene flow can be assumed to be less affected by geographic barriers, but rather by social, ecological, and evolutionary constraints (Rueness et al., 2003). This creates possibilities for assessment of social organization as the cause of local family genetic structuring, independent of the potentially confounding effects of population fragmentation and geographic isolation. In recent decades, several large carnivore species have recolonized parts of their former distribution range and in some areas regained a continuous distribution over large unfragmented landscapes (Chapron et al., 2014).

Due to their different social organization, group‐living and solitary large carnivores may differ in the frequency and strength in which they form long‐lasting family bonds and, more generally, in the rate in which they interact with other individuals during their lifetime. Little is known about the association between social and family genetic structure, especially in solitary large carnivores. Behavioral studies of leopard (*Panthera pardalis*), puma (*Puma concolor*), and tiger (*Panthera tigris*) have revealed that also solitary species can exhibit kin clusters (Elbroch, Quigley, & Caragiulo, 2015; Fattebert et al., 2016; Goodrich et al., 2010; Logan & Sweanor, 2002), but the family genetic structure is less studied.

The Eurasian lynx (*Lynx lynx*) is a solitary predator with a social organization based on territoriality. The species has one of the most widespread distributions of the currently living felids (Breitenmoser et al., 2015). The lynx populations inhabiting Europe differ in their population history and degree of habitat discontinuity (von Arx, Breitenmoser‐Wuersten, Zimmermann, & Breitenmoser, 2004), but in many parts, their populations are highly fragmented (Kaczensky et al., 2013). Thus, it is often challenging to determine the influence of social, ecological, and evolutionary constraints on genetic relatedness and family genetic structure, as this ideally requires high‐resolution genetic data from a continuous and unfragmented population. For the lynx family, studies on kin clusters and philopatry have given inconclusive results. A link between kin structure and dispersal has been found for bobcat (*Lynx rufus*) (Croteau, Heist, & Nielsen, 2010; Janečka et al., 2006), but not for Canada lynx (*Lynx canadensis*) (Campbell & Strobeck, 2006). In Sweden, telemetry studies showed that about one third of Eurasian lynx female offspring remained philopatric (Samelius et al., 2011), indicating the potential for geographic clustering of female relatives. Schmidt, Davoli, Kowalczyk, and Ettore (2016) found an insignificant pairwise geographic genetic distance relationship in a small isolated population of lynx in Białowieza, Poland, but it remains unknown if these results can also be found in other, continuous populations on larger scales. In Latvia, a genetic approach was used to identify the number and location of family groups based on parent–offspring relationships, but details on family genetic structure were not provided (Bagrade et al., 2016).

In this study, we have investigated the genetic relatedness and family structure of Eurasian lynx females in southern Finland. The Eurasian lynx is the only felid species in Finland and the population has shown a substantial population recovery and range expansion during late 1990s and early 21st century (Chapron et al., 2014). The population estimate is based on family group counts, method modified from that in use in Scandinavia (Andrén et al., 2002; Linnell et al., 2007), and has increased from 1,100 to 2,700 adult individuals during years 2007 to 2015 (Luke, 2017), corresponding to an average yearly increase of 12% (Lambda = (2,700/1,100)^1/8^ = 1.12). Distribution area covers whole of Finland, with highest densities in south and central parts of the country (details in: Holmala, 2013). In this continuously distributed population, we analyzed the genetic family structure using 23 autosomal microsatellite markers and tested for possible spatial genetic family structure using a combination of Bayesian clustering, spatial autocorrelation, and forensic genetic parentage analysis. This allowed us to study how genetic relatedness and family structure are organized in space in female lynx unconstrained by low population size, isolation or fragmentation.

## 2 MATERIALS AND METHODS

### 2.1 Study area

**Figure 1:**
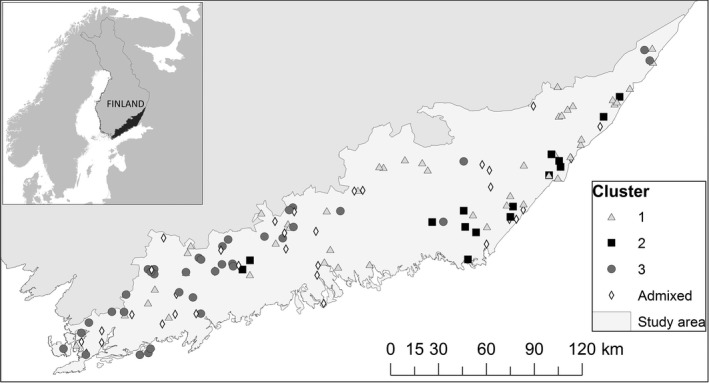
Location of the study area, locations of the Eurasian lynx female tissue samples from years 2007 to 2015, and their identified clusters in southern Finland. Symbols denote the genetic cluster each sample was assigned to cluster 1 = gray triangle; cluster 2 = black square; cluster 3 = dark gray circle; A (admixed) = hollow diamond. *N* = 132

Our study area encompasses two game management districts in the southeastern and eastern parts of Finland, which have a total area of 22,936 km^2^ (Figure 1). About 70% of the terrestrial area is covered by forest, where pine, spruce, and birch are the most common trees. Roughly 10% of the region is covered by lakes. Together with mires, farm land, and urban areas, these two regions form a heterogeneous mosaic of different land uses.

### 2.2 Sampling and DNA extraction

We collected tissue samples from 133 female lynx that had either died naturally (4), in traffic accidents (15) or hunted legally (114) under derogation licenses (granted by the Finnish Wildlife Agency and the Ministry of Forestry and Agriculture) during 2007 to 2015 in Finland (Figure 1). Lynx carcasses were sent to the Taivalkoski Research Station of the Natural Resources Institute Finland (Luke), where the tissue samples were collected and frozen down immediately. Ages of the individuals were determined from cementum annuli analysis of tooth samples by the Matson Laboratory, Milltown, Montana (Matson, 1981).

DNA was extracted from tissue samples with the DNeasy Tissue kit (Qiagen) in the laboratory of Norwegian Institute of Bioeconomy Research, NIBIO—Svanhovd. Samples were analyzed with 23 microsatellite (Short‐tandem repeats; STRs) markers, 20 of which were originally identified for the domestic cat (*Felis catus*; Menotti‐Raymond et al., 1999), whereas the remaining three markers were developed specifically for the Canada lynx (*L. canadensis*) (Lc106, Lc109, Lc110; Carmichael, Clark, & Strobeck, 2000). Additionally, gender identification was performed using the markers on the zinc finger region on the X‐ and Y‐chromosome developed for felids (Pilgrim, McKelvey, Riddle, & Schwartz, 2005). The markers were chosen based on their use in several population genetic studies about lynx in northern Europe (e.g., Hellborg et al., 2002; Rueness et al., 2003; Schmidt, Kowalczyk, Ozolins, Männil, & Fickel, 2009; Ratkiewicz et al., 2014; Rueness, Naidenko, Trosvik, & Stenseth, 2014). Markers were combined into seven multiplex sets (Table 1), and amplification was performed with 1 µl template DNA in a 10 µl PCR containing 5.0 µl 2x Multiplex PCR MasterMix (Qiagen), 1.0 µl Primermix, 0.05 µl BSA (NEB), and 2.95 µl ddH2O. Primer concentration and composition of the different multiplex sets are given in Table 1. The PCR thermal profile was as follows: 10 min at 95°C followed by 29 cycles of 30 s at 94°C, 30 s at 56/57/58/59°C (annealing temperature varied according to multiplex set, see Table 1), 1 min at 72°C, with a final elongation step of 45 min at 72°C. Samples were analyzed on an ABI PRISM 3730 sequencer, and genotyping was performed with GeneMapper v4.1 (Applied Biosystems). Tissue samples were generally analyzed once; however, in order to assess genotyping reliability, 10% of the samples chosen randomly were analyzed a second time.

**Table 1:** 23 different STR markers and the XY‐test used in the genetic analysis. Markers are ordered according to the multiplex set they were assigned to, including the respective annealing temperatures (AT) and fluorescent label (Flag). Final concentration of the respective markers was the same for both forward and reverse primer

Multiplex set	AT (°C)	Marker	Flag	Concentration (µM)
1	58	Fca090	FAM	0.2 µM
		Fca149	VIC	0.1 µM
		Fca723	FAM	0.2 µM
		Fca082	FAM	0.1 µM
2	58	Fca559	NED	0.3 µM
		Fca275	FAM	0.2 µM
		Fca293	FAM	0.2 µM
3	58	Lc110	FAM	0.1 µM
		Fca123	FAM	0.3 µM
		Fca001	FAM	0.4 µM
4	59	Fca567	FAM	0.2 µM
		Fca026	FAM	0.2 µM
		Fca078	FAM	0.3 µM
		Fca031	FAM	0.3 µM
5	58	Fca043	FAM	0.15 µM
		Fca045	NED	0.15 µM
		Fca008	FAM	0.15 µM
		F115	FAM	0.4 µM
6	57	Lc106	FAM	0.5 µM
		Fca126	NED	0.1 µM
		Lc109	FAN	0.5 µM
		Fca391	NED	0.1 µM
7	56	Fca077	FAM	0.2 µM
		XY zinc finger	VIC	0.05 µM

### 2.3 Family genetic structure, spatial autocorrelation, and genetic parentage analysis

We used STRUCTURE v.2.3.4 (Falush, Stephens, & Pritchard, 2003; Pritchard, Stephens, & Donnelly, 2000) to test for indications of family genetic clustering among the females. We set the maximum number of populations to 10 (*K* = 10) with 10 independent runs for each *K* and assuming population admixture and correlated allele frequencies. Burn‐in period was 100,000 Markov‐Chain‐Monte‐Carlo (MCMC) iterations, with a subsequent sampling of 1,000,000 MCMC iterations. We processed the results with Structure Harvester (Earl & von Holdt, 2012), which implements the ad hoc approach of Evanno, Regnaut, and Goudet (2005), and determined the number of putative family genetic clusters, using a membership value of *q* ≥ 0.7 as a threshold value (Kopatz et al., 2014; Pelletier, Obbard, Mills, Howe, & Burrows, 2012). Based on cluster assignment, we performed a factorial correspondence analysis (FCA) among all genotypes, and calculated the number of alleles, observed and expected heterozygosity using GenAlEx 6.5 and inbreeding coefficient using Genetix 4.05.2 (Belkhir, Borsa, Chikhi, Raufaste, & Bonhomme, 1996–2004).

We performed a spatial autocorrelation analysis using GenAlEx 6.501 (Peakall & Smouse 2006; Peakall & Smouse 2012) to examine the relationship between genetic and spatial distance among each pair of female lynx. In the analysis, the family genetic structuring indicated by STRUCTURE was taken into account by using a multipopulation approach. To strike the balance between sample size and spatial resolution, a distance class of 15 km was chosen which corresponds roughly to the largest known female home ranges (i.e., radius) in southern Finland based on telemetry data (K. Holmala unpublished). For each distance class, statistical significance was inferred if the 95% CI around *r* (the average relatedness) did not contain 0, and if *r* exceeded the 95% CI around the null hypothesis of *r* = 0, that is no spatial structure.

We used the program Familias 3.1.9.5 (Egeland & Mostad, 2000; Kling, Tillmar, & Egeland, 2014) to reconstruct parenthood and sib ship from the microsatellite data by calculating likelihood ratios (LRs) for mother–offspring and sibling relationship. The Familias software is used worldwide by forensic laboratories and has been applied to numerous cases, for example resolving family relations and individual identification after disasters. Familias is programmed to recognize several different relationships and makes a pairwise comparison with all individual genotypes against each other and calculates an LR for each relationship. This makes an objective way to distinguish between the most likely relationships. The LR represents the probability of hypothesis one (candidate female is the true mother) divided by the probability of hypothesis two (candidate female is unrelated to the offspring in question) (Marshall, Slate, Kruuk, & Pemberton, 1998).

Based on the a priori probability of being related or unrelated equals to 0.5, the LR shows which relationship is more likely than others. This means a LR of 20 from the genetic analysis corresponds to a 95% probability for relatedness and would be considered as significant support for the relationship in question, while a LR value above 100 would be considered as highly significant support (99% probability of relatedness). Calculating family relationships requires allele frequency data of the population in question and a kinship correction. These we deduced from the data of this study. The Familias software is used worldwide by forensic laboratories and has been applied to numerous cases, for example resolving family relations and individual identification after disasters. In a second step, we used the pairwise geographic distances between death locations of each pair of individuals to calculate the mean and median distance between female parent–offspring, siblings, and all individuals. We also expected that individuals within pairs identified as close relatives would assign to the same family cluster, which was checked against the STRUCTURE results.

## 3 RESULTS

### 3.1 Microsatellite genotyping

We obtained a full genetic profile of 23 STRs for 132 samples (*N* = 133). One sample did not contain enough DNA for successful amplification with the employed STR markers. All of the samples genotyped successfully received a unique identity. For 13 samples, analysis of one or more markers had to be repeated due to amplification failure in the first round, when three tissue samples showed no amplification success in one or two markers.

### 3.2 Genetic diversity

Genetic diversity was relatively high, and expected heterozygosity and observed heterozygosity were almost identical, both averaging 0.66 across all markers (Table 2). Number of alleles varied between 3 and 10 across all 23 markers. There was no evidence of inbreeding in the population, with F_IS_ values not deviating significantly from 0 and averaging 0.012 across all markers and individuals. Tests for HWE followed by Bonferroni correction showed no significant deviation from Hardy–Weinberg equilibrium for any of the markers, except for the marker Fca 559.

**Table 2:** Genetic variation in the studied female lynx population from southern Finland (*N* = 132) during the years from 2007 to 2015. Expected (*H*_E_) and observed (*H*_O_) heterozygosity, number of different alleles (*N*_A_), and inbreeding values (*F*_IS_) calculated for the 23 short‐tandem repeats. Loci deviating significantly from Hardy–Weinberg equilibrium are highlighted by bold *F*_IS_ values

Locus	*N*_A_	*H*_E_	*H*_O_	*F*_IS_
Fca90	4	0.57	0.55	0.044
Fca723	7	0.54	0.58	−0.063
Fca082	5	0.72	0.72	0.012
Fca149	3	0.32	0.30	0.051
Fca567	5	0.77	0.82	−0.069
Fca026	7	0.72	0.72	−0.006
Fca078	5	0.77	0.79	−0.023
Fca031	8	0.80	0.78	0.021
Fca043	5	0.74	0.80	−0.074
Fca045	3	0.30	0.29	0.040
F115	10	0.83	0.79	0.048
Fca008	4	0.72	0.74	−0.028
Lc106	5	0.74	0.74	0.008
Lc109	8	0.78	0.79	−0.011
Fca126	7	0.74	0.77	−0.029
Fca391	3	0.55	0.50	0.090
Fca275	8	0.80	0.77	0.045
Fca293	3	0.60	0.61	−0.006
Fca559	5	0.67	0.61	**0.098**
Lc110	5	0.60	0.55	0.074
Fca123	6	0.71	0.70	0.013
Fca001	8	0.70	0.69	0.010
Fca077	4	0.58	0.52	0.108
Mean	5.6	0.66	0.66	0.012
*SD*	1.9	0.14	0.15	

### 3.3 Fine‐scale genetic family structure

**Figure 2:**
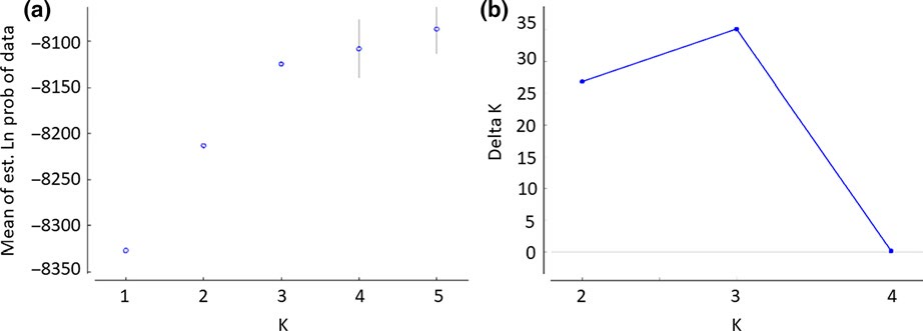
(a) Mean of estimated log‐likelihood values, and (b) rate of log‐likelihood values for 132 Eurasian lynx females for different number of clusters from the software STRUCTURE (Pritchard et al., 2000) postprocessed with Evanno's approach (Evanno et al., 2005)

**Figure 3:**
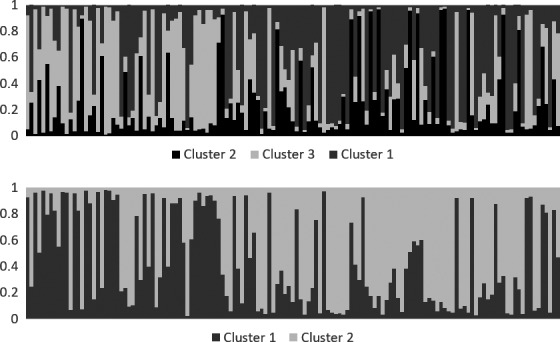
Results from the Bayesian cluster assignment analysis with STRUCTURE of 132 female lynx from southern Finland from 2007 to 2015. Upper panel: model with three clusters; lower panel: model with two clusters. Samples are grouped according to their harvest location and sorted by longitude from west (left) to east (right). The *y*‐axis indicates the membership coefficient *q*, that is the likelihood of belonging to a particular cluster. Each bar represents one individual and the length of each section of one bar corresponds to the *q* value for the respective cluster

**Figure 4:**
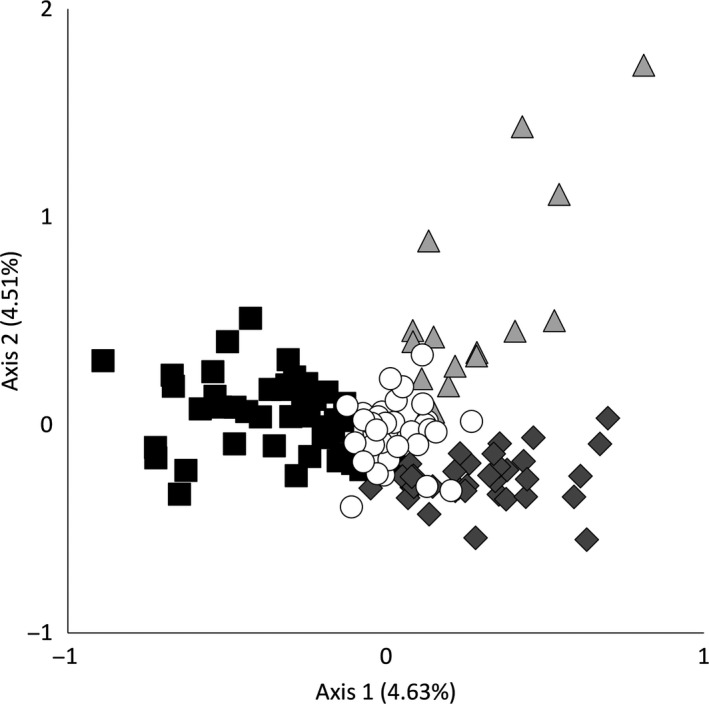
Visualization of the factorial correspondence analysis (FCA) for female lynx genotypes sampled in southern Finland in the time period from 2007 to 2015. Different colors represent the clusters identified by the Bayesian clustering approach: cluster 1 (black squares), cluster 2 (light gray triangles), cluster 3 (gray diamonds), and admixed individuals with a cluster membership value *q* < 0.7 (white circles)

Based on the estimated mean likelihood values and Evanno's Δ*K* (Figure 2b), the STRUCTURE analysis grouped the 132 female lynx individuals in three potential family genetic clusters, which displayed only a slight shift in distribution across the study area (Figure 1). Thirty‐four of 132 lynx could not be assigned to any of the three detected clusters unambiguously. To some extent, Δ*K* supported also an alternative model with only two spatially overlapping family genetic clusters (Figures 2 and 3). Also with this model, the identified clusters displayed only a slight shift in distribution across the landscape (Figure 3), somewhat reminiscent of a geographic–genetic distance relationship. The existence of three potential family genetic clusters across the study area was also supported by an independent FCA, showing three groups for the assigned genotypes and admixed genotypes located among the clusters (Figure 4).

**Figure 5:**
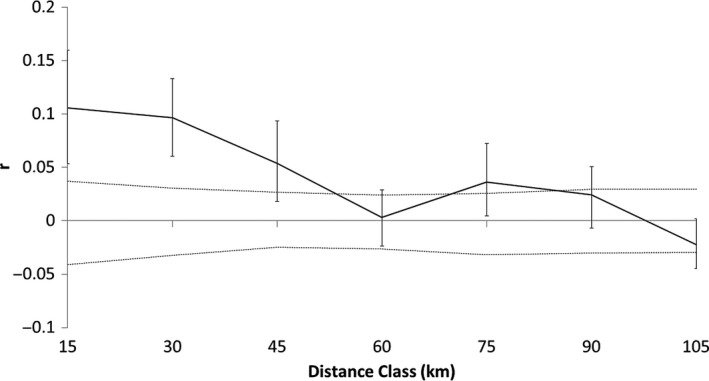
Results of the spatial autocorrelation analysis, that is combined correlation between genetic and spatial distance with GenAlEx 6.501 of lynx samples from southern Finland in the time period 2005 to 2015. The estimated relatedness coefficient (*r*;* y*‐axis) for each distance class (*x*‐axis) is given as a solid line. The 95% confidence interval for the Null hypothesis of random distribution is given as a dashed line, the bootstrap errors are displayed as whiskers. *N* = 132

Consistent with the observed high spatial overlap among the potential family genetic clusters, there was a relatively flat but still highly significant negative correlation between genetic and geographic distance among the 132 female lynx (Figure 5). The average relatedness r of pairs of females was low, but positive and significantly above what one would expect with a random distribution of individuals in all distance classes up to 45 km distance. For the distance class of 60 km, the average relatedness index sunk close to zero, but increased again around 75 km until again falling within the bounds of the 95% CI at the distance classes of 90 km and more.

### 3.4 Genetic parentage analysis

**Figure 6:**
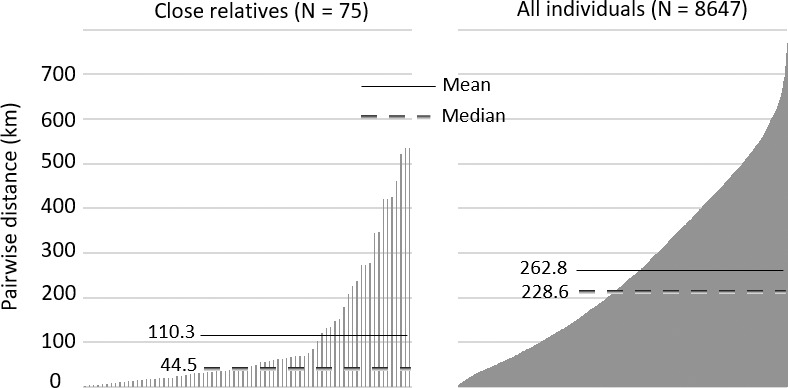
Values for median and mean pairwise geographic distances (km) between individuals of close relatives according to genetic relatedness and all individuals pooled. Median value = dashed line; Mean value =  solid line; *N* = 132

Parentage analyses using the program Familias suggested close, genetic relationships between many different pairs of female lynx: parent–offspring, sibling or half‐sibling, and these pairs of close kin were found both within all three genetic clusters and within the group of admixed individuals (Tables 3, 4, 5). Individuals within pairs of close kin consistently assigned to the same genetic cluster, except in some instances where one of the individuals in a pair was admixed. Among individuals having at least 2‐year difference in age, 32 pairs (47 individuals) were identified as likely mother and daughter by significant support that is a LR above 20 (Table 3). Of these, 97% showed 99% probability of relatedness. Furthermore, 64 pairs (79 individuals) showed LR >20 for being siblings and of these, 68% showed 99% probability of relatedness. In total, 26 pairs (42 individuals) showed LR >20 in both of the analyses above and thus identified as both likely mother–daughter and likely sisters (Table 5). In all of these cases, LR was higher for mother–daughter than for sister, which was also supported by a relatively large age difference in most cases. Assuming that all of these were mother–daughter relationships, the total number of significant sister relationships would be reduced to 38 (57 individuals; Table 4), and the total percentage of individuals with at least one close kin (mother–daughter and sister–sister) would be 63.6% (84 ind.), with 36.4% (48 ind.) being unrelated to all others. The median geographic distance between identified mother–offspring pairs was 36.8 km (mean distance 112.6 km; mean age difference 5.3 years) and between siblings 55.6 km (mean distance 108.6 km; mean age difference 2.5 years). In comparison, the pairwise median geographic distance between all females was 228.6 km (mean distance 262.8 km; mean age difference 3.03 years; Figure 6).

**Table 3:** Recognized 32 Mother–offspring relationships (47 individuals) in a lynx population in southern Finland during the years 2007–2015. The individuals have at least a 2‐year age difference. The likelihood ratios (LR) above 20 were sorted from the highest to the lowest. Individual identification contains ID‐number, year of birth, year of death, and genetic cluster (1, 2, 3, and A = admixed)

Individual 1	Individual 2	Age difference	LR	Distance (km)
FiLL060_2008_2012_2	FiLL075_2000_2013_2	8	4,556,980.00	11.99
FiLL004_2005_2007_1	FiLL041_2000_2011_1	5	2,335,580.00	35.17
FiLL044_2011_2012_3	FiLL086_2006_2013_3	5	650,720.00	13.49
FiLL071_2009_2012_2	FiLL075_2001_2013_2	9	470,696.00	13.32
FiLL084_2013_2013_3	FiLL086_2006_2013_3	7	232,597.00	3.49
FiLL091_2012_2014_1	FiLL130_2005_2015_1	8	151,655.00	278.58
FiLL007_1997_2008_1	FiLL053_2007_2012_1	9	144,159.00	59.38
FiLL007_1997_2008_1	FiLL099_2001_2014_1	3	110,972.00	17.65
FiLL051_2009_2012_1	FiLL058_2012_2012_1	2	106,152.00	19.24
FiLL060_2008_2012_2	FiLL112_2011_2014_2	3	101,015.00	534.65
FiLL049_2004_2012_3	FiLL074_2011_2013_3	6	63,841.50	43.93
FiLL013_2004_2008_A	FiLL045_2003_2012_A	2	21,391.80	69.01
FiLL030_2008_2011_2	FiLL067_2006_2012_2	2	19,333.80	103.98
FiLL091_2012_2014_1	FiLL127_2014_2014_1	2	13,378.30	272.93
FiLL034_2010_2011_2	FiLL067_2006_2012_2	4	11,748.10	6.74
FiLL001_2004_2006_A	FiLL031_2001_2011_1	4	7,866.37	69.92
FiLL101_2013_2014_3	FiLL113_2010_2015_3	4	5,882.27	460.77
FiLL016_2006_2008_3	FiLL113_2010_2015_3	3	3,574.93	421.46
FiLL011_1994_2007_A	FiLL017_2007_2009_A	12	3,547.81	9.38
FiLL004_2005_2007_1	FiLL050_2011_2012_1	5	3,531.88	56.99
FiLL045_2002_2012_A	FiLL059_2012_2012_1	9	3,259.75	1.51
FiLL049_2004_2012_3	FiLL088_2011_2013_3	7	2,382.41	33.71
FiLL040_2009_2011_A	FiLL076_2012_2013_A	2	2,245.33	62.05
FiLL008_2005_2008_1	FiLL118_2014_2014_1	9	1,712.52	19.45
FiLL046_2002_2012_A	FiLL125_2012_2015_A	9	1,662.39	38.07
FiLL063_2011_2012_3	FiLL101_2014_2014_3	2	1,008.62	35.47
FiLL066_2012_2012_A	FiLL068_2008_2012_A	4	961.77	9.39
FiLL063_2011_2012_3	FiLL113_2010_2015_3	2	869.96	426.24
FiLL018_2008_2009_1	FiLL041_2000_2011_1	8	766.41	30.35
FiLL021_2008_2010_A	FiLL046_2003_2012_A	6	333.77	5.57
FiLL097_2012_2014_3	FiLL113_2010_2015_3	3	148.53	420.63
FiLL012_2007_2008_A	FiLL066_2012_2012_A	5	35.61	19.58

**Table 4:** Recognized 38 sibling relationships (60 individuals) in a lynx population in southern Finland during years 2007–2015. The likelihood ratios (LR) above 20 were sorted from the highest to the lowest. Individual identification contains ID‐number, year of birth, year of death, and genetic cluster (1, 2, 3, and A = admixed)

Individual 1	Individual 2	Age difference	LR	Distance (km)
FiLL019_2007_2009_3	FiLL039_2008_2011_3	1	4,583,100.00	31.29
FiLL012_2007_2008_A	FiLL068_2008_2012_A	1	468,167.00	25.35
FiLL054_2011_2012_3	FiLL098_2010_2014_3	1	126,768.00	4.47
FiLL083_2011_2013_2	FiLL089_2010_2014_2	1	111,193.00	24.26
FiLL012_2007_2008_A	FiLL038_2008_2010_1	1	102,532.00	31.47
FiLL129_2011_2015_3	FiLL131_2012_2015_3	1	65,264.70	226.12
FiLL111_2012_2015_A	FiLL120_2013_2014_A	1	42,453.00	131.72
FiLL036_2003_2010_1	FiLL130_2004_2015_1	1	37,306.90	345.29
FiLL038_2008_2010_1	FiLL068_2008_2012_A	0	21,747.40	6.35
FiLL060_2008_2012_2	FiLL071_2009_2012_2	1	12,468.80	15.59
FiLL006_2006_2007_2	FiLL067_2006_2012_2	0	9,763.53	49.72
FiLL115_2014_2014_A	FiLL118_2014_2014_1	0	7,762.84	57.53
FiLL008_2005_2008_1	FiLL115_2014_2014_A	9	6,694.10	76.45
FiLL093_2012_2014_1	FiLL106_2012_2014_1	0	3,286.93	207.87
FiLL023_2010_2010_2	FiLL103_2010_2014_2	0	2,617.23	27.54
FiLL011_1994_2007_A	FiLL119_2005_2015_3	11	1,524.69	121.27
FiLL029_2008_2011_1	FiLL114_2013_2015_1	5	1,305.12	521.55
FiLL006_2006_2007_2	FiLL023_2010_2010_2	4	575.96	18.38
FiLL094_2013_2014_1	FiLL123_2014_2015_1	1	424.58	180.03
FiLL056_2011_2012_A	FiLL079_2011_2012_3	0	364.74	15.59
FiLL034_2010_2011_2	FiLL075_2000_2013_2	10	212.87	64.24
FiLL080_2010_2012_3	FiLL129_2011_2015_3	1	173.58	237.91
FiLL016_2006_2008_3	FiLL039_2008_2011_3	2	166.84	47.49
FiLL044_2011_2012_3	FiLL084_2013_2013_3	2	153.13	10.63
FiLL008_2005_2008_1	FiLL127_2014_2014_1	9	106.61	347.79
FiLL021_2008_2010_A	FiLL125_2011_2015_A	3	100.57	32.55
FiLL016_2006_2008_3	FiLL101_2013_2014_3	7	75.83	39.92
FiLL034_2010_2011_2	FiLL060_2008_2012_2	2	69.16	55.62
FiLL002_2006_2007_1	FiLL057_2006_2012_1	0	58.63	39.34
FiLL063_2011_2012_3	FiLL097_2012_2014_3	1	54.92	16.93
FiLL044_2011_2012_3	FiLL078_2009_2013_3	2	53.37	44.52
FiLL035_2009_2010_1	FiLL050_2010_2012_1	1	43.95	29.94
FiLL106_2012_2014_1	FiLL132_2011_2015_A	1	39.94	147.95
FiLL036_2003_2010_1	FiLL091_2012_2014_1	9	33.74	67.80
FiLL071_2009_2012_2	FiLL089_2010_2014_2	1	26.79	63.53
FiLL027_2009_2010_3	FiLL048_2009_2012_A	0	25.69	153.13
FiLL061_2011_2012_3	FiLL131_2012_2015_3	1	23.30	135.42
FiLL002_2006_2007_1	FiLL104_2009_2014_A	3	23.25	85.08

**Table 5:** Recognized 26 pairs of Mother–offspring and siblings in a lynx population in southern Finland during years 2007–2015. The likelihood ratios (LR) above 20 were sorted from the highest to the lowest. Individual identification contains ID‐number, year of birth, year of death, and genetic cluster (1, 2, 3, and A = admixed)

Individual 1	Individual 2	Age difference	LR Mother–offspring	LR Siblings	Distance (km)
FiLL060_2008_2012_2	FiLL075_2000_2013_2	8	4,556,980.00	829,753.00	11.99
FiLL004_2005_2007_1	FiLL041_2000_2011_1	5	2,335,580.00	1,649,680.00	35.17
FiLL044_2011_2012_3	FiLL086_2006_2013_3	5	650,720.00	19,892.80	13.49
FiLL071_2009_2012_2	FiLL075_2000_2013_2	9	470,696.00	2,092.31	13.32
FiLL084_2013_2013_3	FiLL086_2006_2013_3	7	232,597.00	18,472.40	3.49
FiLL091_2012_2014_1	FiLL130_2004_2015_1	8	151,655.00	5,097.38	278.58
FiLL007_1997_2008_1	FiLL053_2006_2012_1	9	144,159.00	6,033.54	59.38
FiLL007_1997_2008_1	FiLL099_2000_2014_1	3	110,972.00	1,577.43	17.65
FiLL051_2009_2012_1	FiLL058_2012_2012_1	2	106,152.00	606.02	19.24
FiLL060_2008_2012_2	FiLL112_2011_2014_2	3	101,015.00	2,889.34	534.65
FiLL049_2004_2012_3	FiLL074_2010_2013_3	6	63,841.50	61,919.40	43.93
FiLL013_2004_2008_A	FiLL045_2002_2012_A	2	21,391.80	85.02	69.01
FiLL091_2012_2014_1	FiLL127_2014_2014_1	2	13,378.30	469.62	272.93
FiLL034_2010_2011_2	FiLL067_2006_2012_2	4	11,748.10	28.47	6.74
FiLL001_2004_2006_A	FiLL031_2000_2011_1	4	7,866.37	210.61	69.92
FiLL101_2013_2014_3	FiLL113_2009_2015_3	4	5,882.27	208.03	460.77
FiLL016_2006_2008_3	FiLL113_2009_2015_3	4	3,574.93	385.07	421.46
FiLL011_1994_2007_A	FiLL017_2006_2009_A	12	3,547.81	214.95	9.38
FiLL004_2005_2007_1	FiLL050_2010_2012_1	5	3,531.88	277.37	56.99
FiLL045_2002_2012_A	FiLL059_2011_2012_1	9	3,259.75	92.53	1.51
FiLL049_2004_2012_3	FiLL088_2011_2013_3	7	2,382.41	576.59	33.71
FiLL040_2009_2011_A	FiLL076_2011_2013_A	2	2,245.33	490.37	62.05
FiLL008_2005_2008_1	FiLL118_2014_2014_1	9	1,712.52	159.55	19.45
FiLL046_2002_2012_A	FiLL125_2011_2015_A	9	1,662.39	161.59	17.93
FiLL063_2011_2012_3	FiLL101_2013_2014_3	2	1,008.62	266.81	35.47
FiLL066_2012_2012_A	FiLL068_2008_2012_A	4	961.77	56.44	9.39

## 4 DISCUSSION

Using three different genetic approaches, we found support for family genetic structure among females of a solitary carnivore, the Eurasian lynx. The potential family groups were represented by spatially overlapping clusters and a flat but negative pairwise genetic–geographic distance relationship. Furthermore, we found evidence that within each of the putative family genetic clusters, there were several different sibling or mother–daughter pairs. Thus, our results suggest that in a spatially unrestricted population of Eurasian lynx, closely related females tend to cluster together geographically, in agreement with the hypothesis that they may form matrilineal assemblages when not constrained by low density and population fragmentation.

Our estimates of genetic diversity of Eurasian lynx from southern Finland are among the highest. In a review, Schmidt, Ratkiewicz, and Konopiński (2011) concluded that the Eurasian lynx has low to moderate genetic variability. Genetic variation was lowest in Scandinavia and, overall, populations displayed high differentiation and fragmentation (Schmidt et al., 2011). The higher variability for Finnish lynx found in our study may be explained by the larger population size, following successful population recovery and a viable contact through exchange with the larger continuous Russian population (Ratkiewicz et al., 2014; Rueness et al., 2014).

Dispersal has been identified as one of the key elements affecting the genetic structuring of populations. Dispersal barriers are clearly not the reason for the observed family genetic clustering, as the clusters were highly intermixed and not spatially separated. Also, no effective geographic or anthropogenic‐associated barriers for lynx are known from southern Finland. Moreover, the fine‐scale genetic clustering found in our study is on a much smaller spatial scale than would normally be considered relevant for studying genetic subpopulations, stretching only to the similar extent as the diameter of several lynx home ranges (K. Holmala unpublished, Linnell et al., 2007), and with siblings or mother–daughter pairs consistently occurring within clusters. Furthermore, Eurasian lynx is capable of dispersing long distances, for example up to 215 km females and to 428 km males (Samelius et al., 2011), thus potentially allowing for strong gene flow across large areas and among subpopulations (Wayne & Koepfli, 1996) when populations persist through the landscape. In support of this, Ratkiewicz et al. (2014) found evidence of active gene transfer between Finnish and Russian lynx populations. Thus, overall it seems likely that the genetic clustering observed in our study is a signature of the many different pairs of close relatives identified within each cluster, but not between them.

The observed significant correlation of genetic and geographic distances, that is the shorter the distance between individuals, the higher the pairwise relatedness, further corroborates this interpretation. Some of the individuals not clearly assigned to clusters and thus characterized as admixed genotypes, had close relatives among those individuals assigned to a genetic cluster. This indicates mixing of the genetic groups and that those individuals had the same mother but probably a different father. Another possible explanation is that the admixed individuals originate from family groups located outside the study area.

The median distance between all females in our study was over four times longer than between siblings (55.6 km) and over six times more than between parent and offspring (36.8 km). There was a large variation in distances especially for siblings, whereas most mother–daughter pairs were relatively close (although some outliers were found). The cost of defending a given territory may increase with population density, resulting in increased home‐range overlap (Rodgers et al., 2015). Under these conditions, it may be that related individuals tolerate the costs of sharing resources due to benefits gained from inclusive fitness (Anderson, 1989). Kin clusters that are associated with home‐range overlap could potentially support higher local population densities also for Eurasian lynx. However, a strong kin cluster in an area may also potentially hinder immigrating unrelated female lynx from establishing new territories in the vicinity. Immigration by many species of territorial mammals and birds appears to be limited by crowding (Lambin, Aars, & Piertney, 2001). Inversely density‐dependent dispersal, impeding both immigration and emigration, seems to be true for Eurasian lynx in fragmented populations (Zimmermann, 2004; Zimmermann, Breitenmoser‐Würsten, & Breitenmoser, 2005, 2007 ), but whether or not this is also the case in unfragmented ones, such as in Finland, remains to be investigated.

Our results support the hypothesis that Eurasian lynx form matrilineal assemblages at regional scale. However, Elbroch et al. (2015) found mixed support for the existence of cougar matrilineal lines in the southern Yellowstone. Some resident females immigrated into the study area from elsewhere, even while the pedigree revealed several clear matrilineal lines and even some philopatric males. For leopards, when species density increased after decreased harvest pressure, females formed matrilineal kin clusters, suggesting substantial negative effects of harvest disturbances on population size and social structure (Fattebert et al., 2016).

When studying social organization based on genetic data, special attention should be given in selecting the right combination of methods, taking into account species, population size, dispersal capability, the degree of population fragmentation, and the spatial and temporal extent of the study. These may be the reasons why a previous study on a small, isolated population showed generally lack of a relationship between the spatial distance and relatedness among individuals, but on the other hand, showed the domination of the entire population by a limited number of reproducing individuals, which partly indicates kin clustering (Schmidt et al, 2016). It could also mean that social structure is flexible and changes with external conditions. Philopatry, however, does not necessarily lead to genetic clustering (Biek et al., 2006). Biek et al. (2006) found that even though female pumas remained philopatric, there was no genetic clustering, although genetic legacy of females with high reproduction success could be traced. They assumed that either the females were not successful in leaving philopatric offspring or the males, which emigrated from more distant populations, brought enough different alleles to outweigh the clustering phenomenon. It is also worthwhile to ask, is genetic clustering of related individuals always a proof of philopatry? The question relates to the ecologically meaningful spatial scale of the species and the scale used in the study. In species with a social organization determined by family bonds, the spatial distribution of related individuals and the level of genetic clustering we observed might potentially be higher in small, isolated, and fragmented populations as a result of lower dispersal possibilities. Indeed, subadults female lynx may be less prone to cross barriers such as highways and densely populated valleys than males (Zimmermann, Breitenmoser‐Würsten, & Breitenmoser, 2005, 2007 ). However, the opposite effect is also not unthinkable based on the inversely density‐dependent dispersal behavior of lynx (Zimmermann, Breitenmoser‐Würsten, & Breitenmoser, 2005, 2007 ) and, moreover, the possibility that matrilineal structure might disappear at low population densities, as observed for leopard (Fattebert et al., 2016).

The whole Finnish lynx population is of native origin and historically, it experienced periods of population decline. However, it is continuous and well connected to the population in Russia via extensive woodlands (Chapron et al., 2014). As such, our study contributes reference values for genetic parameters from a large lynx population in an almost unfragmented habitat. Robust reference values from large lynx populations are required for the assessment of the genetic status, management, and remedy of the still small reintroduced lynx populations in central Europe (e.g., Zimmermann, 2004; Zimmermann, Breitenmoser‐Würsten, & Breitenmoser, 2005; Zimmermann, Breitenmoser‐Würsten, & Breitenmoser, 2007). Whether the patterns observed among female lynx in our study represent true matrilineal assemblages will be further clarified by including males in analysis. This will also lead to obtaining estimates for the genetic parameters of the whole population. Studies including also males are now needed to further assess the social organization of this species.

## CONFLICT OF INTEREST

None declared.

## AUTHOR CONTRIBUTIONS

KH, SBH, AH, AK, JS, and HGE designed the study. KH was responsible for collected samples. JS generated the raw data in the laboratory. AK, SBH, and JS analyzed the data. All authors contributed to the writing of the manuscript. All authors approved the final version of the manuscript.

## DATA ACCESSIBILITY

Data for this study are available at Dryad Digital Repository: https://doi.org/10.5061/dryad.7pc6ms3 Data files: Holmala_lynx_kinclusters.
